# Supplementary material for: MetaRibo-Seq measures translation in microbiomes
Source: Nat Commun. 2020 Jun 29;11:3268. doi: 10.1038/s41467-020-17081-z (PMC7324362; doi:10.1038/s41467-020-17081-z)
Supplement: Supplementary file 10 — Supplementary Data 7 [file 41467_2020_17081_MOESM10_ESM.zip › File2/Confidence_High_Taxonomy/155664_out.krona.html]

Javascript must be enabled to view this page.

members
magnitude
magnitudeUnassigned
count
unassigned
taxon
rank

155664\_out

55

superkingdom
2
55

phylum
55
1239

55
186801
class

order
186802
55

541000
54
family

species
1898205

SRS011134\_contig\_number\_contig-100\_3397.247551SRS011302\_contig\_number\_20923SRS013476\_contig\_number\_17914SRS013687\_contig\_number\_22722SRS014923\_contig\_number\_20854SRS015431\_contig\_number\_77779SRS015579\_contig\_number\_contig-100\_419.106355SRS015694\_contig\_number\_20429SRS015854\_contig\_number\_23335SRS016095\_contig\_number\_26361SRS016335\_contig\_number\_29040SRS017103\_contig\_number\_contig-100\_25880.25881SRS017701\_contig\_number\_contig-100\_14480.84685SRS017916\_contig\_number\_12078SRS018351\_contig\_number\_445SRS018836\_contig\_number\_16233SRS019068\_contig\_number\_112083SRS019445\_contig\_number\_35188SRS019496\_contig\_number\_16529SRS019808\_contig\_number\_contig-100\_267.80727SRS020233\_contig\_number\_42893SRS021948\_contig\_number\_27724SRS022609\_contig\_number\_contig-100\_1160.246348SRS023914\_contig\_number\_6280SRS024132\_contig\_number\_36528SRS024331\_contig\_number\_23704SRS024435\_contig\_number\_39119SRS024663\_contig\_number\_14760SRS052697\_contig\_number\_11095SRS053214\_contig\_number\_contig-100\_4767.82073SRS053649\_contig\_number\_17395SRS054352\_contig\_number\_22075SRS056273\_contig\_number\_3665SRS063190\_contig\_number\_contig-100\_1585.43723SRS075773\_contig\_number\_21861SRS075821\_contig\_number\_22190SRS076929\_contig\_number\_contig-100\_1960.137226SRS078419\_contig\_number\_33382SRS098571\_contig\_number\_18383SRS098644\_contig\_number\_22585SRS098881\_contig\_number\_17058SRS1041031\_contig\_number\_contig-100\_16047.48948SRS1041092\_contig\_number\_15818SRS104693\_contig\_number\_6142SRS104912\_contig\_number\_contig-100\_9449.86817SRS142923\_contig\_number\_contig-100\_872.137940SRS143148\_contig\_number\_21911SRS143417\_contig\_number\_23121SRS143895\_contig\_number\_27959SRS147346\_contig\_number\_2360SRS148159\_contig\_number\_56860SRS148196\_contig\_number\_contig-100\_33971.33971SRS148784\_contig\_number\_15053SRS893383\_contig\_number\_24270
54

1
186806
family

1
1730
genus


SRS1055069\_contig\_number\_contig-100\_10217.10218
1262878
1
species
